# Supplementary material for: Energy expenditure and body composition changes after an isocaloric ketogenic diet in overweight and obese men: A secondary analysis of energy expenditure and physical activity
Source: PLoS One. 2019 Dec 9;14(12):e0222971. doi: 10.1371/journal.pone.0222971 (PMC6901216; doi:10.1371/journal.pone.0222971)
Supplement: S1 File — (PDF) [file pone.0222971.s001.pdf]

Data Sources and Handling

Abbreviations

$EE_{chamber}$, total daily energy expenditure measured in metabolic chambers; EE_{DLW}, average energy expenditure measured by doubly labeled water; $EE_{nonchamber}$, average energy expenditure on days subjects were living on the ward outside metabolic chambers measured by doubly labeled water; KD, low-carbohydrate/high-fat ketogenic diet; SEE, sleeping energy expenditure.

Data Sources and Handling (see Flowchart below for additional information)

Replication of calorimetry data

Data from Hall et al.'s Table 2 [1] for re-calculation and analysis of were extracted from the "Intake," "chamber," "BC" (body composition) and "DLW" (doubly labeled water) tabs of the Hall et al. dataset published on the Open Science Framework website [2]. In keeping with the original analyses, *P* values were not corrected for multiplicity in this replication or in the analyses below, which may limit inferences when multiple comparisons are made. Similarly, due to the small sample size, no multivariable model was applied to adjust for potential confounders.

Non-chamber EE_{DLW}

Data to calculate Equation (1) and all other analyses of EE_{DLW}, $EE_{nonchamber}$, and $EE_{chamber}$ were taken from the "DLW" tab in the Hall et al. dataset. EE_{DLW} values in Equation 1 correspond to the "TEE DLWChamber unadjusted" values in the "DLW" tab, which were derived using respiratory quotient measured in the chambers during the EE_{DLW} measurement periods. $EE_{chamber}$ values in the "DLW" tab correspond to the "EE binned" values in the "Chamber" tab of the dataset averaged over the four chamber days during the 14-day EE_{DLW} measurement periods.

$EE_{nonchamber}$ for each participant was also calculated using the difference between CO_2 production rates measured in the chambers and by doubly labeled water ($rCO_{2nonchamber}$) according to the following equation:

$$rCO_{2nonchamber}\,(L) = \frac{(rCO_2 \times 22.4 \times 14) - (TCO_2 \times .001)}{10}$$

Where rCO_2 is the daily production rate of CO_2 (mol/d), corresponding to "rCO_2Redo values in the "DLW" tab of the Hall et al. dataset, which is multiplied by the molar volume of gas (22.4) and the number of days in the DLW measurement period. TCO_2 is the total CO_2 production (in ml) measured by respirometry during the four days participants were housed in chambers, which were multiplied by 0.001 to convert to liters. These CO_2 values were derived from "TVCO2"

data in the “chamber” tab of the data set. The product of these calculations was divided by 10 to determine the daily volume of rCO_2 (in L) produced during the 10 non-chamber days.

To calculate $EE_{nonchamber}$ (kcal/d) during the BD period we used Equation 4 from Hall et al. [1] substituting $rCO_{2nonchamber}$ for rCO_2 as below:

$EE_{nonchamber} = \left[\frac{3.85}{RQ} + 1.07\right] \times rCO_{2nonchamber}\,(L)$We used Hall et al.’s [1] Equation 5, which corrects for ketone body excretion (K_{excr} taken from “ketone_excr” values in the “DLW” tab of the dataset), to calculate $EE_{nonchamber}$ during the KD period:

$$EE_{nonchamber} = \left[\frac{3.85}{RQ} + 1.07\right] \times rCO_{2nonchamber}\,(L) - (3.85 \times 0.32 + 1.39) \times K_{\text{excr}}\,(g)$$

RQ is respiratory quotient and for both equations corresponds to the “RQ_Chamber” values in the “DLW” tab of the dataset.

EE_{DLW} outlier

For Subject 04-012, the first of the two body weight measurements associated with body composition assessments during the KD period was performed the day before the “Dose Date” for doubly labeled water (as indicated in the dataset “DLW” tab). The second was performed 12 days later, two days before the end of the EE_{DLW} measurement period. Body weights taken during body composition assessment (“BodyMass_kg”) were extracted from the “BC” (body composition) tab of the dataset and, for the KD EE_{DLW} measurement periods, from “DailyBW” tab of the dataset. Energy intake data were taken from the “EI” tab in the dataset.

Accelerometer data

Accelerometer counts were extracted from the “Accelerometer” tab in the dataset using the “Dose Date” for doubly labeled water in the dataset “DLW” tab as the first day of the EE_{DLW} measurement periods. We included data only from those days during which accelerometer wear time exceeded 720 minutes (12 hours) as specified in the Hall et al. code for analysis of the fractional difference in counts during chamber and non-chamber days.

Sleeping energy expenditure

SEE data were extracted as “SMR Chamber unadjusted” values from the “DLW” tab of the Hall et al. dataset. These values correspond to the “SMR binned” values in the “Chamber” tab of the dataset averaged over the chamber days during the EE_{DLW} measurement periods described above. As in Hall et al. [1], SEE (as kcal/d) was extrapolated from sleeping metabolic rate (as kcal/min).

References

1. Hall KD, Chen KY, Guo J, Lam YY, Leibel RL, Mayer LES, et al. Energy expenditure and body composition changes after an isocaloric ketogenic diet in overweight and obese men. Am J Clin Nutr 2016;104: 324–33.

2. Hall KD. Effect of a Eucaloric Ketogenic Diet on Energy Expenditure: A Pilot Study. Individual Summary Data and SAS Code; 2017. Database: Open Science Framework [Internet]. Available from: https://osf.io/6srfq/

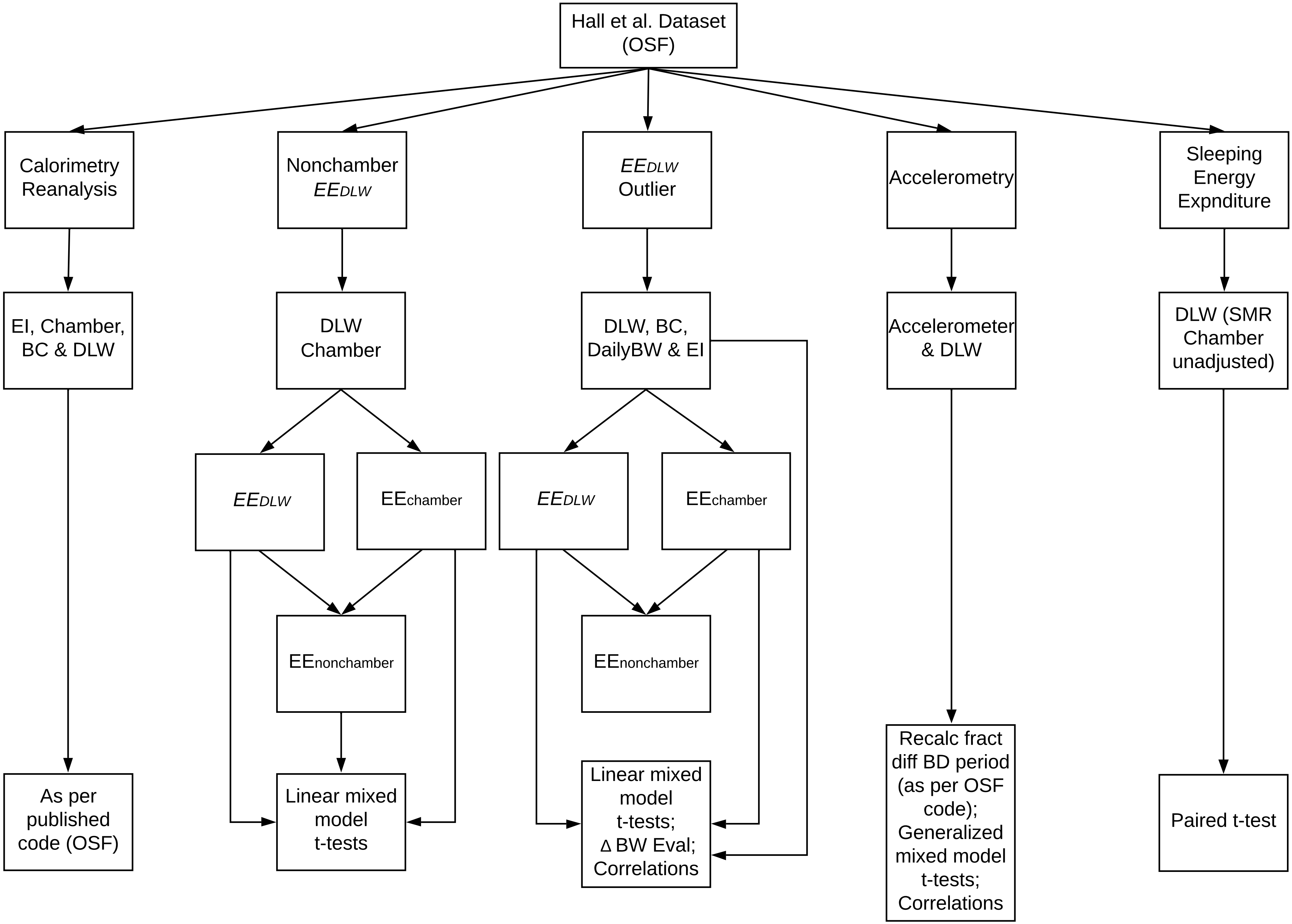

Hall et al. Dataset (OSF)
Calorimetry Reanalysis
Nonchamber EE_{DLW}
EE_{DLW} Outlier
Accelerometry
Sleeping Energy Expnditure
EI, Chamber, BC & DLW
DLW Chamber
DLW, BC, DailyBW & EI
Accelerometer & DLW
DLW (SMR Chamber unadjusted)
EE_{DLW}
$EE_{chamber}$
EE_{DLW}
$EE_{chamber}$
$EE_{nonchamber}$
$EE_{nonchamber}$
As per published code (OSF)
Linear mixed model t-tests
Linear mixed model t-tests; Δ BW Eval; Correlations
Recalc fract diff BD period (as per OSF code); Generalized mixed model t-tests; Correlations
Paired t-test
