# Supplementary material for: Energy expenditure and body composition changes after an isocaloric ketogenic diet in overweight and obese men: A secondary analysis of energy expenditure and physical activity
Source: PLoS One. 2019 Dec 9;14(12):e0222971. doi: 10.1371/journal.pone.0222971 (PMC6901216; doi:10.1371/journal.pone.0222971)
Supplement: S3 File — (PDF) [file pone.0222971.s003.pdf]

Hall et al. 2019: Evaluation of Outlier Selection

Restricted Selection

The protocol for the original Hall et al. study [1], which is the subject of their recent analysis [2], did not specify criteria or methods for identification and handling of outliers. In both studies, subjects were considered outliers, and their energy expenditure data excluded from analysis, based on *post hoc* observations indicating that their energy expenditure measured using doubly labeled water (EE_{DLW}) was not commensurate with other parameters of energy balance. In their recent reanalysis of the original study, two outliers so identified were reported to have gained weight during the ketogenic diet (KD) period despite EE_{DLW} in excess of energy intake (EI). One of the two participants ("Subject A") also gained weight during the BD period although EE_{DLW} exceeded energy intake and showed a "sleight" gain in fat mass during both periods. The other participant ("Subject B") was also identified statistically as an outlier with respect to the magnitude of his increase in EE_{DLW} after the switch from the BD to KD diet. We examined the database for other participants who showed changes in body weight that were discrepant with respect to the difference between their EE_{DLW} and EI because this was a criterion for outlier status that was met by both Subject A and B.

Subjects A and B (Subject ID #'s 04-006 and 04-012, respectively; Group 1 in Table A) showed the greatest increase in EE_{DLW} over EI associated with weight gain of all 17 study participants.

Table A. Putative outliers based on discrepancies between body weight change and the difference in energy expenditure and energy intake.

Group	ID#	EE_{DLW} (kcal/d)	EI (kcal/d)	EE-EI (kcal/d)	ΔBW (kg)
1	04-006[a]	4448	2697	1751	0.6
	04-012[b]	3612	2794	818	0.2
2	02-004	2858	2393	465	1.3
	03-009	2395	2645	-250	-0.2
	04-007	3859	4150	-291	-1.9
3	03-002	2390	2445	-55	-0.4
	03-008	2691	2745	-54	-0.2

[a] and [b] = Subjects A and B, respectively. All measures are during the ketogenic diet (KD) period. EE_{DLW} and EE, energy expenditure by doubly labeled water; EI, energy intake; ΔBW, change in body weight during body composition assessments.

However, five other participants had changes in body weights during the KD period that were incommensurate with the difference between their EE_{DLW} and EI. Three participants (Group 2;

Table A) showed more moderate, but substantial, differences between EE_{DLW} and EI. In two of these, body weight decreased despite an EE_{DLW} that was less than their EI, whereas the third gained weight although EE_{DLW} exceeded EI. Two additional participants (Group 3; Table A) exhibited small, negative differences between EE_{DLW} and EI that were associated with a decrease in body weight.

Subjects A and B showed the two greatest increases in EE_{DLW} and nonchamber energy expenditure ($EE_{nonchamber}$) after the switch from the BD to KD of all 17 participants. Therefore, exclusion of their expenditure data would be expected to reduce any increase in average expenditures after the switch whether adjusted for energy balance or not. Figure A shows the effect of excluding these two participants (Group 1) on average $EE_{nonchamber}$, a primary outcome

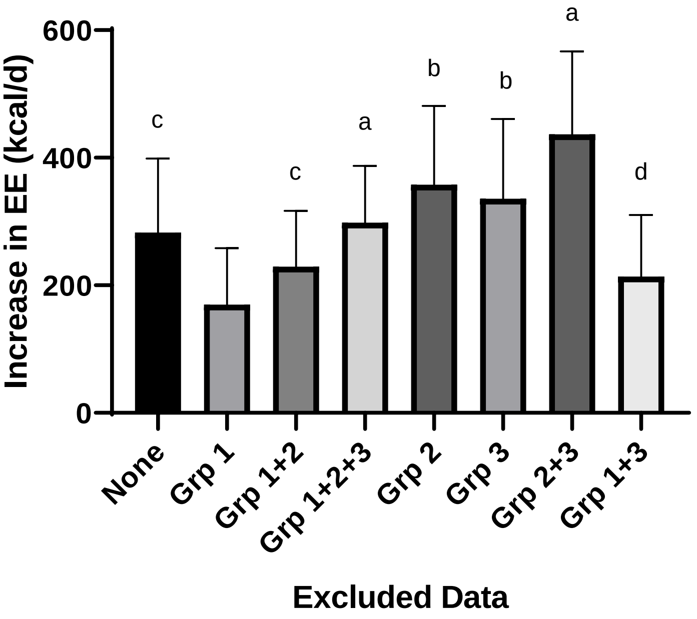

Figure A. Effect of excluding putative outlier data on the increase in non-chamber energy expenditure by doubly labeled water ($EE_{nonchamber}$) after switching from a basal to a ketogenic diet. Values are mean $\pm$ SE. a, b, c, and d = $P < 0.01$, 0.02, 0.03, and 0.05, respectively, by paired t-test. For Group 1, $P = 0.076$.

of our analysis, along with the effect of excluding other outliers (Groups 2 and 3) listed in Table A either separately or in combination with those in Group 1.

Although $EE_{nonchamber}$ increased significantly after the switch to the KD when all 17 participants are included in the analysis, the increase was not statistically significant when data from Subjects A and B (Group 1) were excluded from the analysis. In contrast, the increase of $EE_{nonchamber}$ was significant despite removal of other groups of putative outliers either alone or in combination

with other groups. The effect of excluding Group 2 alone or in combination with Group 1 is especially notable because the energy imbalances reflected by the differences between EE_{DLW} and EI, while less than that in Group 1, were substantial. As reported in this paper (Table 1), the increase in $EE_{nonchamber}$ after the diet switch was statistically significant after exclusion of Subject B's (ID# 04-012) data from the analysis. When only Subject A's data was removed from the analysis, the increase in $EE_{nonchamber}$ after the diet switch (257 $\pm$ 116 kcal/d) was also statistically significant (*P* =0.49 by paired t-test).

Asynchronous Measurement Intervals

The reported gain of body weights during the KD period of Subjects A and B was based on body weight measurements taken during two body composition assessments in the latter part of the diet period. Hall et al. [2] claimed that the interval for body composition assessments was coincident with the EE_{DLW} measurement period. However, inspection of dates in the original study's dataset for DLW dosing and body composition assessments shows this was not the case; body composition and EE_{DLW} measurements periods were coincident in only 6 and 4 out of 17 participants in the BD and KD periods, respectively.

Because the dataset includes dates and daily body weight measurements for all subjects throughout the study, it is possible to determine the change in body weight over the EE_{DLW} measurement period independently from the body weight measures taken during body composition assessments.

As discussed in this paper, Hall et al. [1] reported that Subject B gained 0.2 kg during the KD EE_{DLW} measurement period based on body composition assessments (Table B), but daily body weight measurements show a body weight loss of 0.5 kg during the actual EE_{DLW} measurement period (Table B). Similarly, according to the database, Subject A gained 0.6 kg of weight as per

Table B. Changes in body weight in putative outliers as a function of measurement interval during the ketogenic diet period.

		BC Period	BWBC (kg)	BWCD (kg)	DLW period	BWDD (kg)
**Subject A**	Start	26-Mar-14	63.9	64.5	28-Mar-14	64.6
	End	9-Apr-14	64.5	65.0	10-Apr-14	64.1
	**diff**	--	**0.6**	**0.5**	--	**-0.5**
**Subject B**	Start	18-Jun-14	88.6	89.1	19-Jun-14	89.0
	End	30-Jun-14	88.8	89.1	2-Jul-14	88.5
	**diff**	--	**0.2**	**0.0**	--	**-0.5**

BC, body composition assessment dates; **DLW**, doubly labeled water measurement dates; **BWBC**, body weight for body composition measurement dates using body composition assessment dates; **BWCD**, body weight for body composition measurement dates using daily body weight measurements; **BWDD**, body weight for doubly labeled water measurement dates using daily body weight measurements; **diff**, change in body weight from start to end.

body composition assessments, but according to daily body weight measurements, lost 0.5 kg during the EE_{DLW} measurement period.

No other participants showed such a reversal of body weight change from gain to loss (or vice versa) during the KD period when body weight measurements were synchronized with the EE_{DLW} measurement period, although one participant did so during the BD period. The changes in body weight of Subjects A and B over the EE_{DLW} measurement period measured using daily body weight data were well within one standard deviation of that for the group as a whole (-0.9 ± 0.6 kg, mean $\pm$ SD). The differences between body weight measurements from body composition assessments and recorded daily body weights do not appear to be due to differences in the precision of measurement under the two conditions because changes in body weight over the two body composition assessments as determined using daily body weight measurements were consistent with those measured during composition evaluations (Table B).

References

1. Hall KD, Chen KY, Guo J, Lam YY, Leibel RL, Mayer LES, et al. Energy expenditure and body composition changes after an isocaloric ketogenic diet in overweight and obese men. Am J Clin Nutr 2016;104: 324–33.

2. Hall KD, Guo J, Chen KY, Leibel RL, Reitman ML, Rosenbaum, M, et al. Methodologic considerations for measuring energy expenditure differences between diets varying in carbohydrate using the doubly labeled water method. Am J Clin Nutr 2019;109: 1328-34.
